# Counterconditioning as Treatment to Reduce Nocebo Effects in Persistent Physical Symptoms: Treatment Protocol and Study Design

**DOI:** 10.3389/fpsyg.2022.806409

**Published:** 2022-06-13

**Authors:** Simone Meijer, Henriët van Middendorp, Kaya J. Peerdeman, Andrea W. M. Evers

**Affiliations:** ^1^Health, Medical and Neuropsychology Unit, Leiden University, Leiden, Netherlands; ^2^Leiden Institute for Brain and Cognition, Leiden, Netherlands; ^3^Department of Psychiatry, Leiden University Medical Center, Leiden, Netherlands; ^4^Medical Delta Healthy Society, Leiden University, Technical University Delft, & Erasmus University Rotterdam, Leiden, Netherlands

**Keywords:** nocebo effects, counterconditioning, persistent physical symptoms, open-label, classical conditioning

## Abstract

Persistent physical symptoms have a high prevalence and a large impact for patients and society. To date, treatment effects for these symptoms are often limited. Nocebo effects (i.e., negative outcomes that are not attributable to active treatment components) have a substantial influence on treatment success and can be established via learning through classical conditioning. Therefore, interventions aimed at reducing nocebo effects by means of counterconditioning, in which an alternative association (inhibiting the previous association) is learned, could be a promising method for improving physical symptoms. In experimental studies, counterconditioning has been shown promising in reducing experimentally-induced nocebo effects on pain and itch. Application of counterconditioning procedures to reduce nocebo effects on clinical symptoms has yet to be researched. This paper provides a protocol of a 6-week counterconditioning intervention aimed at reducing nocebo effects and clinical pain in patients with fibromyalgia. A study in patients with fibromyalgia is proposed to examine the feasibility and potential effectiveness of this counterconditioning intervention as a novel treatment method for reducing nocebo effects and generalization to clinical pain symptoms. Results can help design an optimized treatment protocol for reducing nocebo effects, based on the experiences of participants and the first indications of treatment efficacy.

## Introduction

Persistent physical symptoms have a high prevalence and a large impact for patients and society. To date, treatment of these symptoms is effective to a limited extent. Potentially, targeting placebo and nocebo effects (i.e., positive and negative treatment outcomes not attributable to active treatment components, respectively) could provide a novel pathway to prevent or decrease persistent physical symptoms. Placebo and nocebo effects have consistently been found to affect physical symptoms, such as pain, in a positive or negative way, respectively (Benedetti et al., 2003; Colloca et al., 2008; Vase et al., 2009; Colloca and Finniss, 2012; Bartels et al., 2014; Colagiuri et al., 2015; Peerdeman et al., 2016; Ba̧bel et al., 2017; Thomaidou et al., 2020). For example, when patients are told a certain procedure will cause a stinging pain, they may experience more pain than when patients are told the procedure will only feel like a slight pinch. Or, when patients had several treatments at the hospital causing nausea, they might already start to feel nauseous upon merely entering the hospital. Because it is not always possible to prevent such nocebo effects from occurring, it is relevant to examine the potential effects of treatments aimed at reducing nocebo effects.

Classical conditioning is an important associative learning mechanism for the induction of nocebo effects (Bräscher et al., 2018; Thomaidou et al., 2020). During nocebo conditioning, an aversive stimulus (unconditioned stimulus, US; e.g., a highly painful stimulus) leading to an unconditioned response (UR; e.g., pain increase) is paired with a neutral stimulus (typically a sham treatment, such as a sham electrode). Repeated pairing of the two stimuli during a learning or acquisition phase (e.g., pain stimulus together with activation of a sham electrode) can lead to the neutral stimulus (e.g., activation of a sham electrode) becoming a conditioned stimulus (CS). This CS will elicit a similar response (conditioned response; CR, i.e., pain increase) as the UR, even without the US being present. This may also occur in patients with persistent physical symptoms, especially since they may have had several negative treatment experiences. For example, if a person experienced side effects to a certain drug in the past, they may experience these side effects again while taking another drug, merely because they were negatively conditioned in the past (Barsky et al., 2002).

As classical conditioning plays such an important role in the induction of nocebo effects, methods attenuating conditioned effects may be promising for reducing nocebo effects. The attenuation of conditioned effects has been studied more extensively in the field of fear and evaluative conditioning than in the field of nocebo research. Conditioned fear responses can be reduced by extinction, during which the CS is no longer presented together with the US. Through this, the association between CS and US decreases, leading to diminishing of the CR (Vervliet et al., 2013). Furthermore, exposure therapy, which is based on the principles of extinction learning by exposing people to fearful situations in a safe way without anything bad happening to them, can effectively treat fear responses (e.g., spider phobia). Exposure therapy has also been shown effective for chronic pain, as it reduced pain catastrophizing, fear of pain, perceived harmfulness of activities, as well as functional disability (Leeuw et al., 2008; Woods and Asmundson, 2008; Glombiewski et al., 2018). Another method to reduce conditioned effects is counterconditioning, during which the US is replaced by a US of opposite valence. For example, if the CS was previously paired with an electric shock, this shock could be replaced during counterconditioning by a monetary reward. This might lead to more beneficial effects than merely stopping the reinforcement of the CS as in extinction. Multiple studies have found counterconditioning to successfully diminish conditioned effects, but results on the superiority of either extinction or counterconditioning within the field of fear and evaluative conditioning are mixed, as shown by a recent review paper and another recent study (Jozefowiez et al., 2020; Keller et al., 2020). Additionally, long-term efficacy is not often measured, but one of the studies did show that during spontaneous recovery and reinstatement tests (a day after fear induction), diminished threat expectancy was found in the counterconditioning group (Kang et al., 2018). However, CS valence did not differ between the counterconditioning and extinction groups. Furthermore, another study showed counterconditioning to result in a short-lived reduction of distress related to the CS+, but this effect disappeared during later test phases (Hendrikx et al., 2021). Therefore, it may be worthwhile to investigate long-term effects of counterconditioning and whether counterconditioning may be more beneficial in preventing relapse than extinction.

In nocebo research in physical symptoms it has been shown that conditioned nocebo effects are relatively resistant to extinction (Colloca et al., 2008; Colagiuri et al., 2015; Colagiuri and Quinn, 2018) and it may therefore be worthwhile to try the more active strategy of counterconditioning for reducing nocebo effects. While studies comparing efficacy of extinction and counterconditioning for reducing nocebo effects are scarce, they consistently showed counterconditioning to be superior to extinction, as counterconditioning can even reverse nocebo effects into placebo effects (Bartels et al., 2017; Thomaidou et al., 2020). This finding is also supported by a recent preprint paper (Meijer et al., 2021). Although these studies are promising, only healthy participants were examined on the experience of acute physical symptoms, and the experiments were done in a single session and in a highly regulated environment, making it difficult to translate these findings to patients with persistent physical symptoms in clinical care.

Based on the existing literature on counterconditioning in fear and evaluative conditioning studies, as well as the limited knowledge base on experimental counterconditioning in nocebo studies, a counterconditioning treatment protocol was developed for application in patients with persistent physical symptoms, in particular patients with fibromyalgia. As to our knowledge no other study investigated a counterconditioning treatment protocol to counteract nocebo effects, it is important to first study the feasibility and potential effectivity of such a treatment. Therefore, the current paper describes both the development and design of a 6-week counterconditioning treatment protocol aimed to reduce (clinical) pain in patients with persistent physical symptoms.

## Methods and Analysis

A 6-week counterconditioning treatment protocol for use in patients with persistent physical symptoms was developed based on previous literature on counterconditioning in fear and evaluative conditioning, as well as the limited literature on counterconditioning in nocebo effects. The treatment consists of 7 weekly sessions (1 intake session and 6 treatment sessions), with 2 follow-up appointments 3 and 6 months after end of treatment. The treatment protocol will be described firstly below. As this treatment protocol has never been tested before, a first study was designed, to test whether patients are able to complete the protocol and whether there are indications for treatment efficacy. This study design will be described after the treatment protocol. When indications for efficacy have indeed been found, a large-scale randomized controlled trial could be conducted, using the same methods as described below (potentially with minor changes based on the results of the first study).

## Design of Treatment Protocol

### Pain Induction

To be able to test the efficacy of counterconditioning, first a nocebo effect is induced in all participants, using open-label nocebo conditioning. To be able to condition a nocebo effect on pain, pain will have to be induced. In studies on nocebo pain conditioning, several pain-induction methods could be chosen. The most commonly used pain-induction methods are the application of thermal and electrical stimuli. Although these pain-induction methods are effective in research settings, when applying (counter)conditioning in pain patients, the choice of US would ideally be based on the clinical symptoms patients are experiencing in daily life. While some patients with chronic pain may experience a burning-like type of pain (e.g., patients with MS), which resembles thermal pain, or visceral pain (e.g., patients with IBS), other patients (such as patients with fibromyalgia) experience a deep-tissue pain, which is more closely resembled by pressure pain (Wolfe et al., 1990; Gracely et al., 2003; Petzke et al., 2003). Therefore, in this study pressure pain is used during the (counter)conditioning procedures.

**FIGURE 1:**
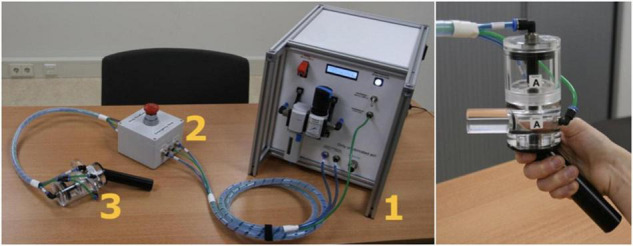
Components of PEPPA. 1 shows the main device, 2 the emergency stop button, and 3 shows the handpiece. The picture on the right shows how the thumb can be placed into the handpiece.

Pressure pain is induced by applying pressure to the thumbnail of the non-dominant hand, with a custom-made automated pneumatic stimulator (named PEPPA), specifically built for this study by SOLO (Support for Research, Laboratories and Education, Leiden University). A handpiece is attached to the stimulator and has a plastic piston that applies pressure via a 1 cm^2^ hard rubber probe. Participants can insert their thumb in a cylinder opening in the handpiece, after which the probe can deliver pressure on the middle of the thumbnail. The thumbnail was selected as a neutral location in which participants feel little to no clinical pain during testing. As previously reported (Petzke et al., 2003; Jensen et al., 2009), this is a safe location for repeatedly delivering pressure stimuli. Stimulus duration is set at 2.5 s, with an inter-stimulus interval of 30 s. The minimal amount of pressure administered is 0.5 kg, whereas the maximum is 13 kg. Finally, an emergency stop is attached to the stimulator, which participants can press if they cannot tolerate the administered pain. After pressing the emergency stop button, all air pressure is released immediately, and they can remove their thumb. All components of PEPPA are displayed in Figure 1.

### Sham Transcutaneous Electrical Nerve Stimulation Device

A sham Transcutaneous Electrical Nerve Stimulation (TENS) device (Bentrotens T37, Bentronic Gesellschaft fuer Medizintechnik GmbH, Wolnzach, Germany) will serve as nocebo conditioning stimulus (CS) in the first part of the treatment (the conditioning session), during which it is associated with an increase of pressure pain. During the second (main) part of the treatment (counterconditioning sessions), it will again serve as CS, now associated with a reduction of pressure pain. This device is developed to automatically switch off after 30 seconds, meaning it will no longer send any electrical pulses and becomes a sham device. The activation within the first 30 seconds is according to usual TENS use (i.e., using mild electrical pulses). Participants are told it is a sham device but are instructed that it will still have an effect on their pain because of the placebo effect as has previously been found to be effective in open-label placebo studies.

### Pressure Pain Calibration

To find the optimal pressure intensity for no pain (0-1/10 NRS), slight pain (2.5-3.5/10 NRS), and moderate pain (5-6/10 NRS), for the individual participant to be used in the intervention, we will conduct a calibration procedure consisting of three phases. For the non-painful stimulus, a minimally painful pressure intensity (0-1/10 NRS) is accepted, as we expect sensitization to occur due to repeated pressure administration. In phase 1 of calibration, pressure stimuli (0.5 kg increments) are applied in ascending order, up to the first pressure intensity participants rated as ≥6. In the second phase, five intensities are applied three times, this time in a random order. The intensities will range from the highest amount of pressure rated as 0 in phase 1, up to the highest pressure rated between 5 and 6. If there will not be any scores between 5 and 6 during phase 1, a formula is used to calculate the best-fitting value (see Supplementary Appendix 1). Then, in the third phase, a calibration check is performed. Three intensities are chosen by taking the median of all intensities that, during the second phase, are rated within the intended ranges for no, slight, and moderate pain. If participants will not score in one or more of the intended ranges, formulas are used to inter- or extrapolate the best-fitting intensity (see Supplementary Appendix 1). During the third phase, participants will need to rate at least one out of two (or two out of three for slight pain) stimuli within the ranges for no, slight and moderate pain. If these requirements will not be met, formulas will again be used to calculate the best-fitting intensity (see Supplementary Appendix 1). Participants who will not experience enough pain (i.e., will not rate their pain at least 5/10 at the maximum amount of pressure), or who will experience too much pain during the lowest intensity (i.e., will rate their pain above 1 from the lowest amount of pressure applied), are excluded.

### Intervention

#### Nocebo Conditioning

**FIGURE 2:**
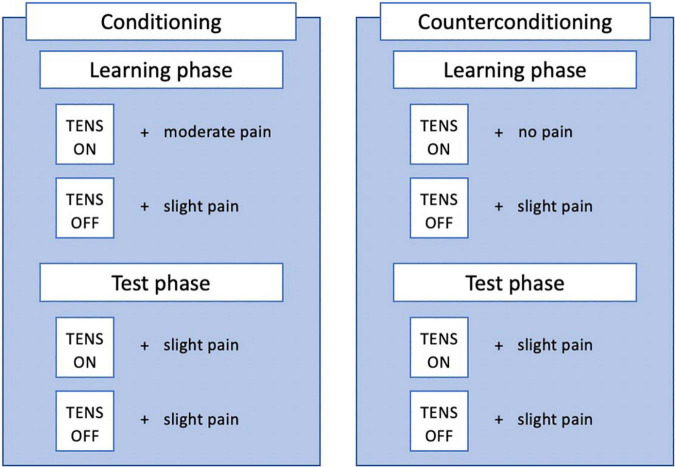
Procedures used for conditioning and counterconditioning.

Nocebo conditioning will consist of a learning and testing phase (see Figure 2). During the learning phase, a message (“ON” or “OFF”) on the screen in either purple or yellow will indicate the activation of the TENS device. If the TENS device is indicated to be on, this is repeatedly paired with a pressure stimulus of a moderate intensity (5-6/10 on NRS). If the TENS device is indicated to be off, this is paired repeatedly with a pressure stimulus of a slightly painful intensity (2.5-3.5 on NRS). The learning phase will consist of 10 experimental (“ON”) trials and 10 control (“OFF”) trials, presented in a pseudorandom order (max 2 stimuli of the same trial type - experimental or control - follow each other). The testing phase will consist of 3 experimental trials and 3 control trials, again in pseudorandom order with the same rule. During the testing phase, a slightly painful stimulus is administered during all trials, regardless of the message on the screen. The test phase is used to assess whether a nocebo effect was induced, as would be indicated by an (on average) higher score on experimental than on control trials. Additionally, participants are given open-label suggestions about the conditioning procedure, as they are told that conditioning is used to teach them that the activation of the sham device increases their pain, by manually increasing the intensity of pressure stimuli during experimental trials. They are also instructed on the nocebo effect and how this will affect their pain. A detailed description of the suggestions can be found in Supplementary Appendix 2.

#### Counterconditioning

Counterconditioning will also consist of a learning and test phase, similar to nocebo conditioning (see Figure 2). Counterconditioning is intended to reduce the nocebo effect, by now repeatedly pairing the alleged TENS activation (ON trials) with a non-painful stimulus, instead of the moderately painful stimulus during nocebo conditioning. The procedure is almost identical to nocebo conditioning, with the only difference being that now a non-painful (0-1/10 on NRS) pressure stimulus is paired with activation of the sham TENS device. The test phase is again used to test whether a nocebo effect is still present, by comparing experimental and control trials. Potentially, a placebo effect could be induced, indicated by an (on average) lower pain score on experimental than on control trials. Additionally, participants are given open-label suggestions about the counterconditioning procedure, as they are told counterconditioning is used to teach them that the activation of the sham device will now decrease their pain, by manually decreasing the intensity of pressure stimuli after experimental trials.

Nocebo effects induced in the lab can be reduced by counterconditioning in a single session (Bartels et al., 2017; Thomaidou et al., 2020). However, in clinical care, patients may have had several negative treatment experiences, instead of a single occasion as in nocebo conditioning in the lab. This may make these nocebo effects even more resistant to treatment. Additionally, as we want to promote long-term efficacy of the treatment, the counterconditioning procedure is repeated several times. As literature on the use of counterconditioning (or related methods, such as systematic desensitization) in clinical care is limited in the field of fear and evaluative conditioning (Keller et al., 2020) and non-existent in nocebo research, it is difficult to determine the optimal number of sessions. Ideally, the treatment is easily accessible and therefore consist of as few sessions as possible, while maintaining optimal treatment efficacy. Therefore, the main intervention during this study will consist of 6 sessions (1 per week) and 2 follow-up sessions (at 3 and 6 months after the final session) to boost the intervention and assess long-term effects. This is much more than previous lab studies on healthy participants. A first study with this design will shed light on the course of nocebo reduction (e.g., how many sessions are needed before the nocebo effect is reduced and after how many sessions a potential effect on clinical pain is found). Potentially, this may also differ per person, as we know susceptibility to nocebo effects also differs between people (Manaï et al., 2019). Based on this information, the amount of sessions may be optimized for future applications. If the nocebo effect is not yet fully reduced after 6 sessions, but a decrease has been found, the intervention could be expanded with more sessions.

#### Open-Label Suggestions & Conditioning

One of the most important disadvantages of using traditional placebo and nocebo conditioning procedures is the fact that it usually involves deception. During traditional placebo and nocebo research, participants are not aware of the fact that the treatment they are receiving is actually a placebo, nor are they aware a conditioning procedure is used (Benedetti et al., 2003; Colloca et al., 2008; Bartels et al., 2014, 2017; Colagiuri et al., 2015; Ba̧bel et al., 2017; Thomaidou et al., 2020, 2020). While deception is generally considered acceptable for research, it is problematic to use deception in clinical care, as patients should always be fully informed regarding the treatment they are about to receive (Riddick, 2003). Deception could harm the trust in both the healthcare professional and the treatment (Miller et al., 2005; Peerdeman et al., 2021), which could lead to reduced treatment efficacy and treatment adherence. Open-label placebos have been examined in several studies (in both healthy and clinical populations) and have been found to be an effective treatment (Sandler and Bodfish, 2008; Kaptchuk et al., 2010; Carvalho et al., 2016; Schaefer et al., 2018; Kleine-Borgmann et al., 2019); in some studies, open-label placebos were even as effective as closed-label placebo treatments (Locher et al., 2017; Lembo et al., 2021). An open-label procedure of nocebo or placebo conditioning has only been compared to closed-label procedures once so far, in which no differences were found between open- and closed-label conditioning (Meeuwis et al., 2019). Although evidence on the efficacy of open-label (counter)conditioning is scarce, closed-label suggestions and (counter)conditioning procedures are not ethically appropriate, and thus open-label counterconditioning seems the most fitting option when considering such procedures for reducing nocebo effects in clinical care. A more detailed description of the suggestions can be found in Supplementary Appendix 2.

### Homework

In between sessions, participants are asked to do homework exercises to promote generalization of nocebo reduction to everyday life. Participants are asked to apply at home what they have learned in the lab, in order to also reduce their clinical pain symptoms. During the first few weeks, participants will use the TENS device at home; they are asked to connect the TENS device to electrodes they will place on their forearm (as during the lab sessions) and are then asked to think back to the lab session and how the device influenced their pain during the session. Then they are asked to imagine the device will now also influence their clinical pain, anywhere in their body. During the final weeks, participants will no longer use the TENS and will instead visualize the function of TENS to reduce their pain. A detailed description of the homework exercises can be found in Supplementary Appendix 3.

### Study Design

#### Participants

Participants are females diagnosed with fibromyalgia syndrome by a general practitioner or medical specialist. Participants must be at least 18 years old and have a good understanding of written and spoken Dutch. Exclusion criteria consist of severe somatic or psychiatric morbidity that may interfere with the study protocol (e.g., heart/lung diseases), Raynaud’s disease, injuries on the non-dominant hand, refusal or inability to remove nail polish or artificial nails on the thumbnail of the non-dominant hand for the experiment, having metal implants in the non-dominant hand or arm, having a medicinal pump, and pregnancy or breastfeeding.

#### Design

**FIGURE 3:**
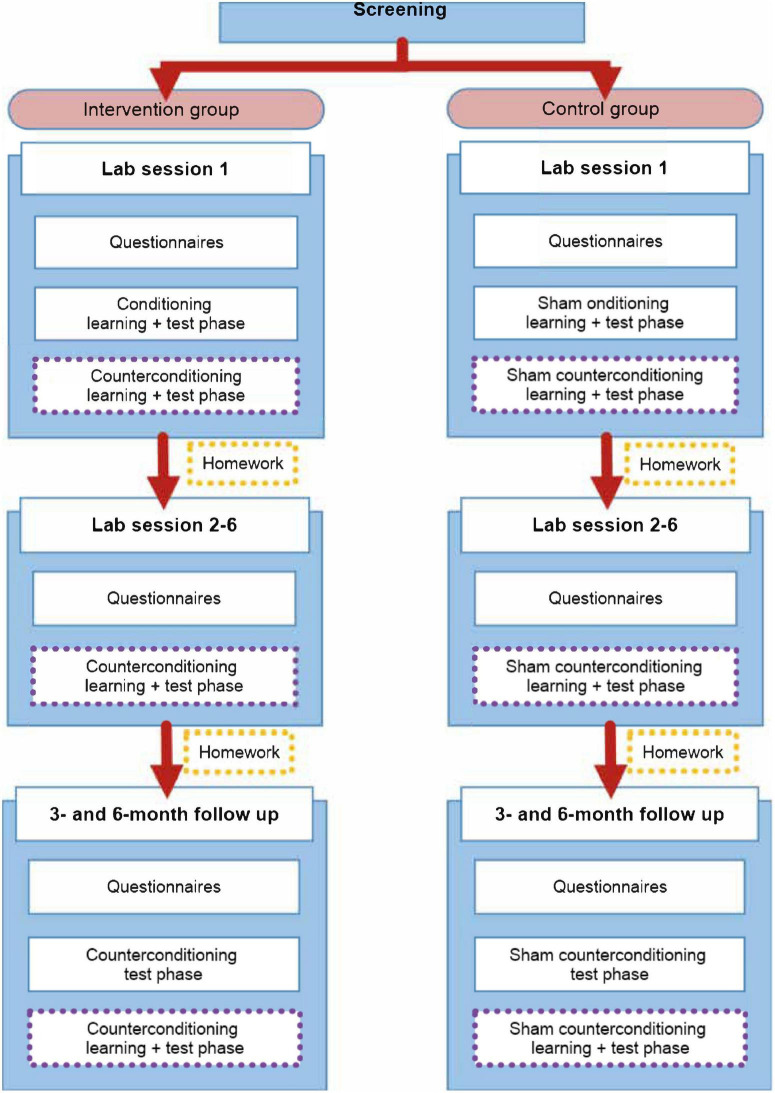
Overview of the treatment protocol for use in patients with persistent physical symptoms, distinguished by group. The (sham) intervention parts of the procedure are emphasized by using purple dotted lines.

A randomized, between-within subjects design is used, with two groups (see Figure 3 below). Participants are randomly assigned (1:1) to either the intervention group or the control group. Participants in the intervention group receives the counterconditioning intervention, whereas participants in the control group receives a sham intervention. A randomization list is made by an independent person and group allocation is noted down on paper and inserted into an opaque envelope, which is opened after the pressure pain calibration procedure, to reduce experimenter bias during calibration. Since all experimental manipulations contain open-label verbal instructions, neither the experimenter nor the participant can be fully blinded to group allocation. Nevertheless, participants are not be explicitly told whether they are in the intervention or control group. The intervention consist of seven weekly sessions and two follow-up sessions (three and six months after the initial seven weeks).

#### Control Group

A sham intervention can serve as control, meaning a sham version of both conditioning and counterconditioning can be used. Sham conditioning and counterconditioning consist of the same amount of trials as the (counter)conditioning procedures, with the same intensities of pain administered (see Figure 2). The major difference is that the pain intensities are not associated with activation or deactivation of the sham TENS device. The 20 pain stimuli of each learning phase are presented randomly, just as the order of the messages (“ON” and “OFF”). Maximally 2 of the same messages (“ON” or “OFF”) follow each other. Furthermore, participants are explicitly told there is no association between the messages and the pain stimuli. More information on the open-label suggestions will be given below and in Supplementary Appendix 2.

As for the homework exercises, participants in the control group also use the TENS device at home during the first weeks, but participants have not learned a specific association between the device and pain relief in the lab. They receive a neutral version of the task. A detailed description of the homework exercises can be found in Supplementary Appendix 3.

#### Self-Report Measures

Several validated questionnaires are filled out by participants, as well as some questions on their medical history and demographics. Table 1 gives an overview of all questionnaires and when they are administered. The Fibromyalgia Impact Questionnaire (FIQ, (Burckhardt et al., 1991), and a Numeric Rating Scale (NRS; 0 (no pain) to 10 (worst pain imaginable)) measuring average intensity of clinical pain during the last week is used to track symptoms of patients throughout the study. Other measures are used to explore the influence of personal characteristics (i.e., Pain Catastrophizing Scale (PCS, (Sullivan et al., 1995)), State and Trait Anxiety Inventory – Trait Scale (STAI-T, (Spielberger, 1983)), State and Trait Anxiety Inventory – State Scale, Short Form (STAI-Ss, (Marteau and Bekker, 1992)), Life Orientation Test Revised (LOT-R, (Scheier et al., 1994))). Additionally, the expectations participants have regarding the intervention are measured every session (except for the intake session). This is measured using 2 questions asking whether participants believe the intervention will influence 1) experimentally-induced pain on the thumb and 2) their clinical pain, using a 0 to 10 numeric rating scale (NRS), with 0 meaning “will not influence pain at all” and 10 meaning “will very strongly influence pain”.

**TABLE 1:** Overview of administered questionnaires throughout the intervention.

Time point	Questionnaire
Intake session	Questions on medical history Questions on demographics State-Trait Anxiety Inventory-Trait Scale Pain Catastrophizing Scale Life Orientation Test-Revised
Session 1-6, 3- and 6-month follow ups	Numeric Rating Scale clinical pain Fibromyalgia Impact Questionnaire State-Trait Anxiety Inventory-State Scale Expectations regarding the intervention
Multiple times during every session (except intake session)	Numeric Rating Scale on pressure pain levels Numeric Rating Scale on valence of CS/control cue
Session 6 + 3- and 6-month follow-ups	Evaluation questionnaire

Experienced pain throughout the sessions (during calibration and (counter)conditioning) is measured using an NRS from 0 to 10 (0 indicating no pain, 10 indicating worst pain imaginable on the hand). Additionally, valence is measured, as it is argued in studies on fear and evaluative conditioning that the change in CS valence might be important to prevent reinstatement of the previously conditioned effects (De Jong et al., 2000; Meulders et al., 2015; Van Dis et al., 2019). It is therefore important to investigate whether CS valence is successfully altered by counterconditioning. Valence of the CS and control cue is measured after the 1^st^ and 10^th^ trial of the learning phase and after the 1^st^ trial of the test phase. It is measured using an NRS ranging from −5 to +5, with −5 meaning “extremely unpleasant”, 0 meaning “neutral” and +5 meaning “extremely pleasant”.

Finally, to measure patient satisfaction, an evaluation questionnaire is filled out by participants at the end of session 6 and the 3- and 6-month follow-up. This questionnaire includes questions on the patient-researcher interaction, their satisfaction regarding the intervention in general, whether the amount of sessions is feasible, whether they believed the intervention to have an effect on their pain (both in the lab and at home) and which group they thought they were placed in.

#### Procedure

Participants are invited to the lab 9 times (intake session, 6 intervention weeks, 3- and 6-month follow-up). Figure 3 gives an overview of the procedure.

##### Screening and Intake

Participants are screened over the phone before inviting them to the lab, to check whether they are eligible to participate. If eligible, participants are invited to the lab for a first appointment, the intake session, in which the intervention is fully explained to the participants. Additionally, participants are asked to fill in most of the questionnaires mentioned in Table 1.

##### Session 1

During session 1, participants fill out several questionnaires (see Table 1). Afterwards, pain levels are calibrated as described above, followed by a 5-min break. Subsequently, participants undergo the nocebo conditioning procedure, which is not repeated in the other sessions. After nocebo conditioning and a 10-min break, the counterconditioning procedure follows. The session is concluded by instructions on the homework exercises, which participants do daily between the sessions. In total, the duration of the first session is approximately 2 h.

##### Session 2-6

During sessions 2 to 6, participants will again fill out several questionnaires. Then pressure pain calibration is checked by only repeating phase 3 of the calibration procedure, to ensure the administered intensities are still perceived similarly to the first session. If this is not the case, intensities are adjusted using standard formulas (Supplementary Appendix 1). After a 5-min break, the counterconditioning procedure will start, after which the session is concluded. In between sessions, participants will do daily homework exercises. The duration of session 2-6 is approximately 35-45 min. Only during session 6, an evaluation questionnaire is filled out by participants at the end of the session.

##### Follow Ups

The follow ups are almost identical to sessions 2-6. Again, the sessions will start with the participants filling out questionnaires, after which calibration step 3 is repeated. Then, participants will undergo only a test phase of counterconditioning, to test long-term effects of the intervention. Through this, it is assessed whether the nocebo effect is still absent and whether potentially a placebo effect is still present. After this test phase, the regular counterconditioning procedure is repeated, to boost the effect of the intervention. In both follow-up sessions, a short evaluation questionnaire is filled out at the end of the session. The duration of both follow-up sessions is approximately 45 min.

#### Objectives and Statistical Analyses

As the current treatment protocol has never been tested before, it would be relevant to first test the feasibility of the protocol. Once proven feasible, a larger randomized controlled trial could be conducted to test efficacy of the treatment protocol.

The main study parameter in a first study is the feasibility of the counterconditioning intervention. This can be done by looking at the drop-out rate; by measuring participants’ satisfaction with the intervention; by examining what, according to the participants, is causing the possible increase and reduction of experimentally-evoked pressure pain in the test phase of (counter)conditioning (e.g., the TENS device, the placebo or nocebo effect); and by examining the amount of experimentally-evoked pressure pain reported during the test phase of counterconditioning, whether this reduces over time, as well as the speed of this reduction.

Next to examining the feasibility, a first study could also exploratively look at indications that the counterconditioning intervention affects experimentally-evoked pressure pain from pre- to post-treatment. To this aim, it can be explored whether the induced nocebo effect in the intervention group can be successfully reduced (or even reversed) by comparing the confidence intervals of the change in the conditioned nocebo effect from the test phase of conditioning (session 1) to the counter conditioned nocebo effect from the test phase of counterconditioning (session 6) in the intervention group and the control group. The effect size and confidence interval of the difference between groups will also be calculated. Additionally, all sessions are compared in terms of nocebo reduction, to be able to assess speed of nocebo reduction.

Confidence intervals of the change in the conditioned nocebo effect from the test phase of conditioning (session 1) to the counter conditioned nocebo effect from the test phase of counterconditioning at 3- and 6-month follow-up in the intervention group and the control group can also be explored. The effect size and confidence interval of the difference between groups are calculated.

Finally, whether there are indications of the influence of personal characteristics (e.g., expectations regarding the intervention, amount of anxiety during testing) on the feasibility of the study and the potential effects of the intervention can be explored. This is done by comparing the confidence intervals of the scores on the different questionnaires on personal characteristics in participants who drop out versus participants who do not drop out and in participants who show a reduction of the nocebo effect versus participants who do not show any treatment effect (within the intervention group).

## Discussion

The aim of this paper was to describe a newly-developed counterconditioning treatment protocol for patients with persistent physical symptoms and its translation into a study design of which a first study could test its feasibility and explore its potential effectiveness. Nocebo effects in clinical care can have a substantial detrimental impact on treatment outcomes but cannot always be prevented, therefore it is important to investigate potential treatment options for reducing nocebo effects. While counterconditioning has been experimentally investigated in healthy participants, the proposed treatment protocol is the first using counterconditioning to reduce nocebo effects in chronic pain patients (e.g., patients with fibromyalgia). A first study will provide important insights on how to potentially further develop this treatment protocol for reducing nocebo effects in clinical practice. Multiple facets are considered, such as patient satisfaction, drop-out rate, and patient characteristics.

### Anticipated Results

#### Feasibility

For a first study, the main outcome parameter is the feasibility of the treatment protocol. Firstly, drop-out rate can give an insight into patient satisfaction and into feasibility of completing the treatment protocol as a patient. While we strive to prevent drop-out as much as possible, one of the main aims of the study is to investigate whether it is feasible for patients to receive this type of treatment weekly, for multiple weeks in a row. As for patient satisfaction, we aim for participants to be satisfied with their treatment, by being fully open about the study procedures (hence the open-label nature of the study). Nevertheless, we are evoking pain in our protocol, which may negatively affect patient satisfaction. Furthermore, although the study is open-label, participants may still be slightly skeptical about the procedures used, as found in other open-label studies (Carvalho et al., 2016; Lembo et al., 2021). We strive to reduce this skepticism as much as possible by providing an explanation of placebo treatments and how they can be efficacious. Furthermore, although patients were skeptical and/or expected better results from active treatments, open-label placebos were still effective in the aforementioned studies. Finally, we expect participants to be able to correctly answer what influenced the intensity of the experimentally-induced pain, which is not the TENS device itself, but the nocebo or placebo effect (induced by (counter)conditioning), as we are fully open about the procedure. Nevertheless, it cannot be ruled out participants may think the TENS device still has some influence on their pain or that they attribute their experiences to other phenomena. If this happens, that might indicate that our instructions regarding the treatment are not sufficiently clear to participants and need adjustment.

#### Efficacy of the Treatment

In a first study, no formal conclusions can be drawn on the efficacy of the treatment. However, we do expect multiple findings will point towards successful induction and reduction of nocebo effects. Firstly, for the treatment group, we expect participants to score higher on experimental (“TENS on”) trials than on control (“TENS off”) trials in the test phase of nocebo conditioning (session 1), which would be in line with other studies on nocebo conditioning (e.g., Benedetti et al., 2003; Colloca et al., 2008, 2010; Bartels et al., 2014; Colagiuri et al., 2015; Thomaidou et al., 2020). For the control group, we do not expect to see a difference between the trial types, similar to results from other studies using a sham conditioning group (Thomaidou et al., 2020). Secondly, we expect the nocebo effect (defined as the average difference between experimental and control trials in the test phase of conditioning) to be larger in the treatment group than in the control group. Thirdly, we expect to find a reduction of the nocebo effect in the treatment group, meaning the nocebo effect is expected to be (close to) zero or even below zero (indicating a placebo effect), during the test phase of counterconditioning in session 6. This is based on the findings of the few studies investigating counterconditioning for reducing nocebo effects of physical symptoms in healthy participants (Bartels et al., 2017; Thomaidou et al., 2020). As for the speed of this reduction, we will explore whether the nocebo effect can be reduced from the first session or starting from later sessions, and whether this reduction is stable throughout the sessions. For the 3- and 6-month follow-ups, we expect to find similar results, indicating potential long-term efficacy of counterconditioning. For the control group, we do not expect to find a change in effect from conditioning (session 1) to counterconditioning (session 6 or follow-ups). We also do not expect to find a placebo effect at the end of session 6 and the follow-ups for the control group.

Then, regarding valence of the CS, we expect the counterconditioning procedure to affect the valence from pre- to post-treatment. During nocebo conditioning (session 1), we expect participants in the experimental group to rate the CS more negatively than at the end of counterconditioning (session 6 and both follow-ups). For the control group, we do not expect to find differences in valence from pre- to post-treatment.

Finally, it can be explored whether the counterconditioning procedure can be translated to clinical pain. As this is the first time this is investigated, it is yet to be determined whether the protocol could be beneficial for reducing clinical pain. We do, however, expect to find the counterconditioning sessions, strengthened by the homework exercises, being able to reduce clinical pain of participants in the treatment group, but not in the control group.

Should the aforementioned results indeed be found, a large-scale randomized controlled trial could be conducted afterward, be able to draw more robust conclusions about the efficacy of the nocebo counterconditioning treatment.

### Anticipated Strengths and Limitations

An important strength of the current treatment protocol is that it is aimed at reducing existing nocebo effects, instead of aimed at preventing nocebo effects. Although arguably the prevention of nocebo effects is crucial in clinical practice, this is not always feasible, as patients need to be informed about potential negative effects of a treatment, which may induce nocebo effects. Consequently, developing a treatment protocol aimed at nocebo reduction can be of added value to clinical practice. Another important strength of this treatment protocol is the fact that an open-label procedure is used. As mentioned before, traditional paradigms using (counter)conditioning are mostly deceptive procedures, which is not suitable for a clinical treatment protocol. As some studies have already shown the efficacy of open-label placebos, as well as open-label conditioning, open-label counterconditioning does seem a promising treatment strategy for reducing nocebo effects.

Using open-label paradigms may have some disadvantages. While it is unethical to use deception in clinical practice, the risk of a response bias may be higher. Participants are explicitly told (counter)conditioning is used to influence their pain and this thus makes the intensity of stimuli more predictable than during traditional paradigms. Furthermore, they may be more aware of the expected outcomes, which could also increase the risk of participants answering in a way fitting the expectations of the researchers. This risk could be minimized optimally by letting the participants answer on a computer, instead of directly answering the researcher. Additionally, nocebo effects are usually not induced in an open-label fashion, as patients are not aware of the fact they are being conditioned. Furthermore, instead of a single type of negative experience, most likely a combination of experiences has induced nocebo effects in patients. While it is important to mimic such a negative experience for all participants, it may be complicated to figure out which stimuli migh be best used for counterconditioning. Therefore, once proven potentially effective, the final counterconditioning protocol may have to be adjusted to account for these differences in patients’ experiences.

Moreover, the effect of extinction and exposure therapy may be context-dependent, as found by several studies (Rodriguez et al., 1999; Mystkowski et al., 2002; Vansteenwegen et al., 2005). Return of fear appears to be higher in people who were submitted to a context change after extinction or exposure than in people remaining within the same context. This may also be the case for the counterconditioning of the nocebo effect, but this has not yet been researched. While in the lab the nocebo effect is induced and reduced within the same context, this is typically not the case in clinical practice, as the treatment occurs in a different context than in which effects were induced. Furthermore, it may prove difficult to translate effects from the lab or clinic to the home situation, as this would again be a change of context. By incorporating homework exercises in between the weekly sessions, we aim to enable generalization of the effects from the lab to other contexts. The exploratory results of the study will give insight into whether this may lead to successful generalization of the effects or whether alternative and/or extra steps in the protocol are necessary to promote generalization.

### Practical Implications

We expect a first study to show the counterconditioning intervention to be feasible and will provide preliminary indications for its effectiveness in relieving pain in fibromyalgia patients. Should this indeed be observed, a large-scale randomized controlled trial could be conducted afterward to assess the efficacy of the counterconditioning protocol. Results of the first study can help inform the design of the final protocol, based on the experiences of participants in the feasibility study and the first indications regarding treatment efficacy. Additionally, the counterconditioning protocol should ideally also be compared to other methods aimed at reducing conditioned responses, to test whether this method is superior over more commonly used procedures, such as extinction (exposure therapy), overshadowing, latent inhibition and blocking (Quinn and Colagiuri, 2018). If shown effective in a larger study, the protocol could potentially be implemented in clinical practice for treatment of nocebo effects in patients with persistent physical symptoms, which could consequently lead to a decrease in these physical symptoms.

## Ethics Statement

This study was reviewed and approved by the Medical Ethical Committee Leiden-Den Haag-Delft (number NL 66812.058.18). Written informed consent was obtained from all participants for their participation in this study. Furthermore, the study has been preregistered in the Dutch Trial Register (NL8189).

## Author Contributions

SM wrote the first draft and all (sub)sections of the manuscript. SM, HM, KP, and AE contributed to the conception and design of the treatment protocol and the manuscript and provided critical feedback on the manuscript. All authors contributed to manuscript revision, read, and approved the submitted version.

## Conflict of Interest

The authors declare that the research was conducted in the absence of any commercial or financial relationships that could be construed as a potential conflict of interest.

## Publisher’s Note

All claims expressed in this article are solely those of the authors and do not necessarily represent those of their affiliated organizations, or those of the publisher, the editors and the reviewers. Any product that may be evaluated in this article, or claim that may be made by its manufacturer, is not guaranteed or endorsed by the publisher.

## References

[B1] Ba̧bel P. Bajcar E. A. Adamczyk W. Kicman P. Lisińska N. Świder K. (2017). Classical conditioning without verbal suggestions elicits placebo analgesia and nocebo hyperalgesia. *PLoS One* 12:e0181856. 10.1371/journal.pone.0181856 28750001 PMC5531508

[B2] Barsky A. J. Saintfort R. Rogers M. P. Borus J. F. (2002). Nonspecific medication side effects and the nocebo phenomenon. *JAMA* 287 622–627. 10.1001/jama.287.5.622 11829702

[B3] Bartels D. J. P. van Laarhoven A. I. M. Haverkamp E. A. Wilder-Smith O. H. Donders A. R. T. van Middendorp H. (2014). Role of conditioning and verbal suggestion in placebo and nocebo effects on itch. *PLoS One* 9:e91727. 10.1371/journal.pone.0091727 24646924 PMC3960153

[B4] Bartels D. J. P. van Laarhoven A. I. M. Stroo M. Hijne K. Peerdeman K. J. Donders A. R. T. (2017). Minimizing nocebo effects by conditioning with verbal suggestion: a randomized clinical trial in healthy humans. *PLoS One* 12:e0182959. 10.1371/journal.pone.0182959 28910291 PMC5598922

[B5] Benedetti F. Pollo A. Lopiano L. Lanotte M. Vighetti S. Rainero I. (2003). Conscious expectation and unconscious conditioning in analgesic, motor, and hormonal placebo/nocebo responses. *J. Neurosci.* 23 4315–4323. 10.1523/JNEUROSCI.23-10-04315.2003 12764120 PMC6741114

[B6] Bräscher A.-K. Witthöft M. Becker S. (2018). The underestimated significance of conditioning in placebo hypoalgesia and nocebo hyperalgesia. *Pain Res. Manage.* 2018:6841985. 10.1155/2018/6841985 29670678 PMC5833150

[B7] Burckhardt C. S. Clark S. R. Bennett R. M. (1991). The fibromyalgia impact questionnaire: development and validation. *J. Rheumatol.* 18 728–733. 1865419

[B8] Carvalho C. Caetano J. M. Cunha L. Rebouta P. Kaptchuk T. J. Kirsch I. (2016). Open-label placebo treatment in chronic low back pain: a randomized controlled trial. *Pain* 157 2766–2772. 10.1097/j.pain.0000000000000700 27755279 PMC5113234

[B9] Colagiuri B. Quinn V. F. (2018). Autonomic arousal as a mechanism of the persistence of nocebo hyperalgesia. *J. Pain* 19 476–486. 10.1016/j.jpain.2017.12.006 29269281

[B10] Colagiuri B. Quinn V. F. Colloca L. (2015). Nocebo hyperalgesia, partial reinforcement, and extinction. *J. Pain* 16 995–1004. 10.1016/j.jpain.2015.06.012 26168876

[B11] Colloca L. Finniss D. (2012). Nocebo effects, patient-clinician communication, and therapeutic outcomes. *JAMA* 307 567–568. 10.1001/jama.2012.115 22318275 PMC6909539

[B12] Colloca L. Petrovic P. Wager T. D. Ingvar M. Benedetti F. (2010). How the number of learning trials affects placebo and nocebo responses. *Pain* 151 430–439. 10.1016/j.pain.2010.08.007 20817355 PMC2955814

[B13] Colloca L. Sigaudo M. Benedetti F. (2008). The role of learning in nocebo and placebo effects. *Pain* 136 211–218. 10.1016/j.pain.2008.02.006 18372113

[B14] De Jong P. J. Vorage I. Van Den Hout M. A. (2000). Counterconditioning in the treatment of spider phobia: effects on disgust, fear and valence. *Behav. Res. Ther.* 38 1055–1069. 10.1016/S0005-7967(99)00135-7 11060935

[B15] Glombiewski J. A. Holzapfel S. Riecke J. Vlaeyen J. W. S. de Jong J. Lemmer G. (2018). Exposure and CBT for chronic back pain: an RCT on differential efficacy and optimal length of treatment. *J. Consult. Clin. Psychol.* 86 533–545. 10.1037/ccp0000298 29781651

[B16] Gracely R. H. Grant M. A. B. Giesecke T. (2003). Evoked pain measures in fibromyalgia. *Best Pract. Res.* 17 593–609. 10.1016/S1521-6942(03)00036-6 12849714

[B17] Hendrikx L. J. Krypotos A.-M. Engelhard I. M. (2021). Enhancing extinction with response prevention via imagery-based counterconditioning: results on conditioned avoidance and distress. *J. Behav. Ther. Exp. Psychiatry* 70:101601. 10.1016/j.jbtep.2020.101601 32835958

[B18] Jensen K. B. Kosek E. Petzke F. Carville S. Fransson P. Marcus H. (2009). Evidence of dysfunctional pain inhibition in Fibromyalgia reflected in rACC during provoked pain. *PAIN®* 144 95–100. 10.1016/j.pain.2009.03.018 19410366

[B19] Jozefowiez J. Berruti A. S. Moshchenko Y. Peña T. Polack C. W. Miller R. R. (2020). Retroactive interference: counterconditioning and extinction with and without biologically significant outcomes. *J. Exp. Psychol. Anim. Learn. Cogn.* 46 443–459. 10.1037/xan0000272 33030955

[B20] Kang S. Vervliet B. Engelhard I. M. van Dis E. A. M. Hagenaars M. A. (2018). Reduced return of threat expectancy after counterconditioning versus extinction. *Behav. Res. Ther.* 108 78–84. 30064009 10.1016/j.brat.2018.06.009

[B21] Kaptchuk T. J. Friedlander E. Kelley J. M. Sanchez M. N. Kokkotou E. Singer J. P. (2010). Placebos without deception: a randomized controlledtrial in irritable bowel syndrome. *PLoS One* 5:e15591. 10.1371/journal.pone.0015591 21203519 PMC3008733

[B22] Keller N. E. Hennings A. C. Dunsmoor J. E. (2020). Behavioral and neural processes in counterconditioning: past and future directions. *Behav. Res. Ther.* 125:103532. 10.1016/j.brat.2019.103532 31881357 PMC6983350

[B23] Kleine-Borgmann J. Schmidt K. Hellmann A. Bingel U. (2019). Effects of open-label placebo on pain, functional disability, and spine mobility in patients with chronic back pain: a randomized controlled trial. *Pain* 160 2891–2897. 10.1097/j.pain.0000000000001683 31479068

[B24] Leeuw M. Goossens M. E. van Breukelen G. J. de Jong J. R. Heuts P. H. Smeets R. J. (2008). Exposure in vivo versus operant graded activity in chronic low back pain patients: results of a randomized controlled trial. *Pain* 138 192–207. 10.1016/j.pain.2007.12.009 18242858

[B25] Lembo A. Kelley J. M. Nee J. Ballou S. Iturrino J. Cheng V. (2021). Open-label placebo vs double-blind placebo for irritable bowel syndrome: a randomized clinical trial. *Pain* 162 2428–2435. 10.1097/j.pain.0000000000002234 33605656 PMC8357842

[B26] Locher C. Nascimento A. F. Kirsch I. Kossowsky J. Meyer A. Gaab J. (2017). Is the rationale more important than deception? A randomized controlled trial of open-label placebo analgesia. *Pain* 158 2320–2328. 10.1097/j.pain.0000000000001012 28708766

[B27] Manaï M. van Middendorp H. Veldhuijzen D. S. Huizinga T. W. Evers A. W. (2019). How to prevent, minimize, or extinguish nocebo effects in pain: a narrative review on mechanisms, predictors, and interventions. *Pain Rep.* 4:e699. 10.1097/PR9.0000000000000699 31583340 PMC6749907

[B28] Marteau T. M. Bekker H. (1992). The development of a six-item short-form of the state scale of the Spielberger State—Trait Anxiety Inventory (STAI). *Br. J. Clin. Psychol.* 31 301–306. 10.1111/j.2044-8260.1992.tb00997.x 1393159

[B29] Meeuwis S. H. Van Middendorp H. Pacheco-Lopez G. Ninaber M. K. Lavrijsen A. P. M. Van Der Wee N. (2019). Antipruritic placebo effects by conditioning H1-antihistamine. *Psychosom. Med.* 81 841–850. 10.1097/PSY.0000000000000743 31490841 PMC6844655

[B30] Meijer S. Karacaoglu M. van Middendorp H. Peerdeman K. J. Veldhuijzen D. S. Jensen K. (2021). Efficacy of open-label counterconditioning for reducing nocebo-effects on pressure pain. *PsyArXiv[Preprint]* 10.31234/osf.io/uhd3s 36932915

[B31] Meulders A. Karsdorp P. A. Claes N. Vlaeyen J. W. S. (2015). Comparing counterconditioning and extinction as methods to reduce fear of movement-related pain. *J. Pain* 16 1353–1365. 10.1016/j.jpain.2015.09.007 26434783

[B32] Miller F. G. Wendler D. Swartzman L. C. (2005). Deception in research on the placebo effect. *PLoS Med.* 2:e262. 10.1371/journal.pmed.0020262 16173830 PMC1198039

[B33] Mystkowski J. L. Craske M. G. Echiverri A. M. (2002). Treatment context and return of fear in spider phobia. *Behav. Ther.* 33 399–416. 10.1016/S0005-7894(02)80035-1 16942960

[B34] Peerdeman K. J. Geers A. L. Della Porta D. Veldhuijzen D. S. Kirsch I. (2021). Underpredicting pain: an experimental investigation into the benefits and risks. *Pain* 162 2024–2035. 10.1097/j.pain.0000000000002199 33470747

[B35] Peerdeman K. J. van Laarhoven A. I. M. Peters M. L. Evers A. W. M. (2016). An integrative review of the influence of expectancies on pain. *Front. Psychol.* 7:1270. 10.3389/fpsyg.2016.01270 27602013 PMC4993782

[B36] Petzke F. Clauw D. J. Ambrose K. Khine A. Gracely R. H. (2003). Increased pain sensitivity in fibromyalgia: effects of stimulus type and mode of presentation. *Pain* 105 403–413. 10.1016/S0304-3959(03)00204-5 14527701

[B37] Quinn V. F. Colagiuri B. (2018). Using learning strategies to inhibit the nocebo effect. *Int. Rev. Neurobiol.* 138 307–327. 10.1016/bs.irn.2018.01.011 29681332

[B38] Riddick F. A. Jr. (2003). The code of medical ethics of the american medical association. *Ochsner. J.* 5 6–10. PMC3399321 22826677

[B39] Rodriguez B. I. Craske M. G. Mineka S. Hladek D. (1999). Context-specificity of relapse: effects of therapist and environmental context on return of fear. *Behav. Res. Ther.* 37 845–862. 10.1016/S0005-7967(98)00106-5 10458048

[B40] Sandler A. D. Bodfish J. W. (2008). Open-label use of placebos in the treatment of ADHD: a pilot study. *Child* 34 104–110. 10.1111/j.1365-2214.2007.00797.x 18171451

[B41] Schaefer M. Sahin T. Berstecher B. (2018). Why do open-label placebos work? A randomized controlled trial of an open-label placebo induction with and without extended information about the placebo effect in allergic rhinitis. *PLoS One* 13:e0192758. 10.1371/journal.pone.0192758 29513699 PMC5841659

[B42] Scheier M. F. Carver C. S. Bridges M. W. (1994). Distinguishing optimism from neuroticism (and trait anxiety, self-mastery, and self-esteem): a reevaluation of the Life Orientation Test. *J. Pers. Soc. Psychol.* 67 1063–1078. 10.1037//0022-3514.67.6.1063 7815302

[B43] Spielberger C. D. (1983). *Manual for the State-Trait Anxiety Inventory (Form Y1 – Y2).* Palo Alto, CA: Consulting Psychologists Press.

[B44] Sullivan M. J. Bishop S. R. Pivik J. (1995). The pain catastrophizing scale: development and validation. *Psychol. Assess.* 7 524–532.

[B45] Thomaidou M. A. Veldhuijzen D. S. Peerdeman K. J. Wiebing N. Z. S. Blythe J. S. Evers A. W. M. (2020). Learning mechanisms in nocebo hyperalgesia: the role of conditioning and extinction processes. *Pain* 161 1597–1608. 10.1097/j.pain.0000000000001861 32149863 PMC7302337

[B46] Van Dis E. A. M. Hagenaars M. A. Bockting C. L. H. Engelhard I. M. (2019). Reducing negative stimulus valence does not attenuate the return of fear: two counterconditioning experiments. *Behav. Res. Ther.* 120 103416–103416. 10.1016/j.brat.2019.103416 31254717

[B47] Vansteenwegen D. Hermans D. Vervliet B. Francken G. Beckers T. Baeyens F. (2005). Return of fear in a human differential conditioning paradigm caused by a return to the original acquistion context. *Behav. Res. Ther.* 43 323–336. 10.1016/j.brat.2004.01.001 15680929

[B48] Vase L. Petersen G. L. Riley I. I. I. J. L. Price D. D. (2009). Factors contributing to large analgesic effects in placebo mechanism studies conducted between 2002 and 2007. *PAIN®* 145 36–44. 10.1016/j.pain.2009.04.008 19559529

[B49] Vervliet B. Craske M. G. Hermans D. (2013). Fear extinction and relapse: state of the art. *Annu. Rev. Clin. Psychol.* 9 215–248. 10.1146/annurev-clinpsy-050212-185542 23537484

[B50] Wolfe F. Smythe H. A. Yunus M. B. Bennett R. M. Bombardier C. Goldenberg D. L. (1990). The american college of rheumatology 1990 criteria for the classification of fibromyalgia. *Arthritis Rheum.* 33 160–172. 10.1002/art.1780330203 2306288

[B51] Woods M. P. Asmundson G. J. (2008). Evaluating the efficacy of graded in vivo exposure for the treatment of fear in patients with chronic back pain: a randomized controlled clinical trial. *Pain* 136 271–280. 10.1016/j.pain.2007.06.037 17716819

