# Supplementary material for: Counterconditioning as Treatment to Reduce Nocebo Effects in Persistent Physical Symptoms: Treatment Protocol and Study Design
Source: Front Psychol. 2022 Jun 13;13:806409. doi: 10.3389/fpsyg.2022.806409 (PMC9237388; doi:10.3389/fpsyg.2022.806409)
Supplement: Supplementary file 1 [file Data_Sheet_1.PDF]

Supplementary materials

Appendix 1: Formulas used during calibration

Ascending series:

If no score between 4.5 and 5.5 was reported during ascending series, the following formula was used to determine the maximum amount of pain administered during random series:

$$\textit{Maximum pain intensity (in kPa)} = kPa_y + \frac{(kPax - kPay)}{(x-y)} * (5 - y)$$

- x = Lowest NRS score higher than desired range (4.5-5.5/10)
- y = Highest NRS score lower than desired range (4.5-5.5/10)
- kPax = kPa intensity at NRS score x (if multiple intensities were scored identically, the highest intensity was chosen).
- kPay = kPa intensity at NRS score y (if multiple intensities were scored identically, the highest intensity was chosen).

Random series & Calibration check:

No pain:

When none of the stimuli was scored between 0-1, a pressure intensity was used of 50 kPa lower than the lowest intensity during random series/calibration check.

Slight pain:

If no score between 2 and 3 was reported during random series, the following formula was used to determine the slight pain value:

$$\textit{Slight pain intensity (in kPa)} = kPa_y + \frac{(kPax - kPay)}{(x-y)} * (2.5 - y)$$

- x= Lowest NRS score higher than desired range (2-3/10)
- y = Highest NRS score lower than desired range (2-3/10)
- kPax = kPa intensity at NRS score x (if multiple intensities were scored identically, the median of those intensity was taken).

- kPay = kPa intensity at NRS score y (if multiple intensities were scored identically, the median of those intensity was taken).

Moderate pain:

If no score between 4.5 and 5.5 was reported during random series (but there were scores **higher** than 5.5), the following formula was used to determine the slight pain value:

$$\textit{Moderate pain intensity (in kPa)} = kPa_y + \frac{(kPax - kPay)}{(x-y)} * (5 - y)$$

- x= Lowest NRS score higher than desired range (4.5-5.5/10)
- y = Highest NRS score lower than desired range (4.5-5.5/10)
- kPax = kPa intensity at NRS score x (if multiple intensities were scored identically, the median of those intensity was taken).
- kPay = kPa intensity at NRS score y (if multiple intensities were scored identically, the median of those intensity was taken).

If no score between 4.5 and 5.5 was reported during random series (but there were scores **lower** than 5.5), the following formula was used to determine the slight pain value:

$$\textit{Moderate pain intensity (in kPa)} = kPa_y + \frac{(kPax - kPay)}{(x-y)} * (5 - x)$$

- x= Highest reported NRS score
- y = Second highest reported NRS score
- kPax = kPa intensity at NRS score x (if multiple intensities were scored identically, the median of those intensity was taken).
- kPay = kPa intensity at NRS score y (if multiple intensities were scored identically, the median of those intensity was taken).

If there are no scores in the intended ranges, the same formulas as for random series can be used.

Appendix 2: Verbal suggestions (translated from Dutch)

Intake session (both groups)

The rationale behind this treatment method is derived from the placebo effect; you may have already heard of this before? In our study, we want to focus on the opposite effect, called the nocebo effect. Nocebo effects are adverse effects caused by the idea that a treatment may have negative consequences. An example is healthy participants receiving an inert pill during research participation, who still experience side effects, even when the pill is inert. This is caused by the information that is provided about the pill, which leads to negative expectations. People may then interpret innocent symptoms that everyone can experience every now and then as side effects from the pill. We also know that nocebo effects can have a big influence on how people are physically feeling and how well they respond to treatment.

Learning plays an important role in the induction of nocebo effects, for example by conditioning. The most well-known example of conditioning is the example of Pavlov's dogs. Pavlov showed that physical responses can be provoked by something neutral, by repeatedly pairing this neutral stimulus to something the body automatically responds to. Dogs will start to salivate when they see and smell food. By ringing a bell repeatedly when they see and smell the food, the dogs start to pair the bell with the food. Eventually, they will start to salivate when only hearing the bell and they have thus learned a new association. This can also happen with people receiving a certain treatment. For example, if people get nauseous each time they receive a certain treatment in the hospital, eventually they may feel nauseous when they enter the hospital, before receiving any treatment. These negative experiences with previous treatments may also lead to future treatments working less well.

We know that learning processes can also play a role in chronic pain. In this study we want to try to influence these learning processes. In the study, we would like to simulate a negative treatment experience; this is needed to teach you a new association, just as with Pavlov's dogs. In our study, we will use a non-functional device. Normally, this device can send small electrical pulses, which can influence pain. As this device is non-functional, we cannot send any electrical pulses.

In the study, we first want to induce a negative association with this device. The new method we want to investigate is that we reduce this learned association again, by using "counterconditioning". With counterconditioning the negative association will be altered to a more positive experience. Afterwards, we want to translate this to daily life.

In this study, there is a 50% chance you will end up in the intervention group, which receives the treatment explained before. There is also a 50% chance of being assigned to the control group. Participants in the control group will receive an inactive version of the method. This control group is really important, to be able to investigate whether there are any indications of the efficacy of the treatment. After the study you will be told which group you were assigned to.

Session 1-6 – Intervention group

Conditioning (nocebo induction, only session 1):

As mentioned in the previous session, physical responses to a treatment can be taught by using conditioning. We know from previous research that an inert pill can cause people to experience more pain. This is called a nocebo effect. These effects can be caused not only by pills, but also by other forms of treatment, such as a device.

In our study we use an inactive device, instead of a pill. Normally, this device can send mild electrical pulses to influence pain. This is an inactive device, meaning we cannot send electrical pulses.

In this part of the study we want to teach you that whenever the device is turned on, your pain will be worse. Everytime the device is turned on, we will give you a pressure stimulus of a higher intensity, compared to when the device is turned off. Because of this you will learn that your pain will be worse when the device is turned on. Eventually, turning on the device will increase your pain.

During the tests, we will administer several pressure stimuli. Before and during each stimulus you will see on the screen whether the device is turned on or off.

Counterconditioning (nocebo reduction):

During this part of the study, we want to reduce the physical response to the device again. You will again receive a series of stimuli. By using counterconditioning, we now want to teach you that whenever the device turns on, your pain will reduce. Each time the device is turned on, I will administer pressure stimuli of a lower intensity, compared to when the device is turned off. You will then learn that the device will reduce your pain and eventually merely turning on the device will reduce your pain.

Session 1-6 – Control group

Sham conditioning (only session 1):

As mentioned in the previous session, physical responses to a treatment can be taught by using conditioning. We know from previous research that an inert pill can cause people to experience more pain. This is called a nocebo effect. These effects can be caused not only by pills, but also by other forms of treatment, such as a device.

In our study we use an inactive device, instead of a pill. Normally, this device can send mild electrical pulses to influence pain. This is an inactive device, meaning we cannot send electrical pulses.

During the tests, we will administer several pressure stimuli. Before and during each stimulus you will see on the screen whether the device is turned on or off. You will receive stimuli of different intensities. The intensity of the stimuli are **not** related to the device being on or off.

Sham counterconditioning:

In this part you will again receive certain pressure stimuli. Before and during each stimulus you will see on the screen whether the device is turned on or off. You will receive stimuli of different intensities. The intensity of the stimuli are **not** related to the device being on or off.

Appendix 3 – Homework exercises

Experimental group:

Before the exercise, participants will attach the TENS electrodes to the TENS and will place them on their forearm (during week 1+2). During week 3 and 4, they will only use the TENS half of the times. During week 5 and 6, the TENS is no longer used. Instead they will visualize using the TENS.

"Think back to the situation in the lab, during which the device was attached to your forearm and during which you received pain stimuli. In the second part of the session, a stimulus of a lower intensity was administered whenever the device was turned on. Try to imagine this situation as good as possible in your head. Focus on how the activation of the device reduced the amount of pain on your thumbnail. Feel how device activation caused less pain. Now imagine the device reducing the pain you are feeling at this moment, anywhere in your body. Try to focus your attention on the reduction of your pain because of the activation of the device."

Control group:

Before the exercise, participants will attach the TENS electrodes to the TENS and will place them on their forearm (during week 1+2). During week 3 and 4, they will only use the TENS half of the times. During week 5 and 6, the TENS is no longer used. Instead they will visualize using the TENS.

"Think back to the situation in the lab, during which the device was attached to your forearm and during which you received pain stimuli. During the session, several pressure stimuli were applied to your thumbnail. Think back to how this felt; focus on the different pressure stimuli administered on your thumbnail. Now focus on what you are feeling at this moment, anywhere in your body."
